# Silencing of the Violaxanthin De-Epoxidase Gene in the Diatom *Phaeodactylum tricornutum* Reduces Diatoxanthin Synthesis and Non-Photochemical Quenching

**DOI:** 10.1371/journal.pone.0036806

**Published:** 2012-05-18

**Authors:** Johann Lavaud, Arne C. Materna, Sabine Sturm, Sascha Vugrinec, Peter G. Kroth

**Affiliations:** 1 Fachbereich Biologie, Universität Konstanz, Konstanz, Germany; 2 UMR7266 ‘LIENSs,’ CNRS/University of La Rochelle, Institute for Coastal and Environmental Research, La Rochelle, France; Rutgers University, United States of America

## Abstract

Diatoms are a major group of primary producers ubiquitous in all aquatic ecosystems. To protect themselves from photooxidative damage in a fluctuating light climate potentially punctuated with regular excess light exposures, diatoms have developed several photoprotective mechanisms. The xanthophyll cycle (XC) dependent non-photochemical chlorophyll fluorescence quenching (NPQ) is one of the most important photoprotective processes that rapidly regulate photosynthesis in diatoms. NPQ depends on the conversion of diadinoxanthin (DD) into diatoxanthin (DT) by the violaxanthin de-epoxidase (VDE), also called DD de-epoxidase (DDE). To study the role of DDE in controlling NPQ, we generated transformants of *P. tricornutum* in which the gene (*Vde*/*Dde*) encoding for DDE was silenced. RNA interference was induced by genetic transformation of the cells with plasmids containing either short (198 bp) or long (523 bp) antisense (AS) fragments or, alternatively, with a plasmid mediating the expression of a self-complementary hairpin-like construct (inverted repeat, IR). The silencing approaches generated diatom transformants with a phenotype clearly distinguishable from wildtype (WT) cells, i.e. a lower degree as well as slower kinetics of both DD de-epoxidation and NPQ induction. Real-time PCR based quantification of *Dde* transcripts revealed differences in transcript levels between AS transformants and WT cells but also between AS and IR transformants, suggesting the possible presence of two different gene silencing mediating mechanisms. This was confirmed by the differential effect of the light intensity on the respective silencing efficiency of both types of transformants. The characterization of the transformants strengthened some of the specific features of the XC and NPQ and confirmed the most recent mechanistic model of the DT/NPQ relationship in diatoms.

## Introduction

Diatoms belong to the most abundant photosynthetic microorganisms on Earth accounting for about 40% of the primary production in the oceans [1]. The ecological success of both planktonic and benthic diatoms is partly owed to their ability to tolerate and quickly acclimate to a rapidly changing light climate [2]–[4]. Growth in fluctuating light intensities requires a fast responding photosynthetic machinery [2], [4], [5] to protect the chloroplast from potential damage by excess energy absorption at saturating light intensities [2], [4], [6], [7]. Plants and algae have evolved a number of photoprotective mechanisms including the non-photochemical quenching of fluorescence, NPQ [2], [4], [6], [7]. NPQ mediates thermal dissipation of excess light energy absorbed by the light-harvesting antenna complex (LHC) of photosystem II (PS II). NPQ is mainly controlled by the inter-conversion of epoxidized to de-epoxidized forms of xanthophyll carotenoids during the so-called xanthophyll cycle (XC) [8]–[10]. The xanthophyll de-epoxidation is mediated by an enzyme, the de-epoxidase, which is located in the lumen of the thylakoids, while the back-conversion is ensured by a stromal epoxidase [8]–[10]. The light-dependent build-up of the transthylakoidal proton gradient (ΔpH) and the subsequent acidification of the lumen is necessary for the binding of the de-epoxidase to the thylakoid membrane in order to get access to its xanthophyll substrate [8], [9]. This process is regulated by the protonation of a glutamic acid-rich domain located in the highly charged C-terminal part of the enzyme and by the protonation of histidine residues located in the lipocalin region [11]–[13]. In diatoms, there are two XCs [9], [10], [14], one of them is identical to the XC found in higher plants, performing the de-epoxidation of violaxanthin (Vx) to zeaxanthin (Zx) via the intermediate antheraxanthin by the violaxanthin de-epoxidase (VDE). The other and main XC of diatoms includes only a single step, the de-epoxidation of diadinoxanthin (DD) into diatoxanthin (DT). The pigments of the Vx cycle are precursors of DD and DT, and of the main LHC xanthophyll, fucoxanthin [9], [10].

In diatoms, genes encoding for de-epoxidases assumed to be responsible for one or both XCs have been found [10], [15], [16]. In *Phaeodactylum tricornutum* as well as in *Thalassiosira pseudonana*, there is a single gene (*Vde*) encoding for a VDE protein, also named DDE (for DD de-epoxidase) with a strong similarity to the VDE of higher plants. Two additional proteins, named ‘VDE-like’ or VDL plus two ‘VDE-related’ or VDR [5] in *P. tricornutum* are only distantly related. While it was observed *in vitro* that the DDE could participate in Vx and the DD cycles [17], the localization and the role of the VDLs remains under debate [10]. It was proposed that the VDLs might participate exclusively in the DD cycle [15] although they have a much less charged C-terminal domain [15], [16]. While VDLs are thus unlikely to be ΔpH-regulated and to be involved in the XCs the same ways as DDE [16], there is so far no experimental evidence that they should not be able to synthesize DT. The xanthophyll de-epoxidation in diatoms additionally shows specific features (see [4], [9]) such as i) a fast activation of the DDE due to its reaction to a low acidification of the lumen, ii) a low requirement of the DDE for its co-factor ascorbate, iii) a need of the DDE for a special composition and arrangement of the lipids of the thylakoid membrane. The presence of DT, together with the acidification of the lumen, is crucial for NPQ development in the light-harvesting complex (LHC) of photosystem II (PSII) [2], [9], [18], [19]. In *P. tricornutum* [20], [21] and other diatom species [22]–[24], the amount of DT synthesized in the light can be high and it strongly correlates with the extent of NPQ. The slope of the NPQ *versus* DT relationship can vary with species and light acclimation [22], [25] and it might be related to the specific structural organization of thylakoids in diatoms [4]. Such a difference is assumed to have ecophysiological and ecological implications. In nature, the XC and the NPQ are of primary importance for the acclimation of diatoms to the fluctuations of the underwater light climate [2], [9], [26], which recently has been described to be an important functional trait that potentially may influence niche adaptation [2], [23], [27]–[30].

In higher plants and in the green alga *C. reinhardtii*, the suppression of VDE was shown to be very useful for gaining new insights into the role of the XC in photoprotection [31]–[33]. Since double-stranded RNA (dsRNA) was proven to be an extremely potent activator of mRNA degradation by RNA interference (RNAi), the experimental introduction of dsRNA into target cells became a powerful tool for functional genomics specifically mediating gene silencing [34]. Experimental introduction of complementary RNA molecules into target cells can be achieved via transgene transcription or micro-injection of small interfering RNAs (siRNA). Although little is known about the mechanisms underlying gene silencing in diatoms, successful suppression of endogenous gene expression by gene silencing was recently demonstrated in *P. tricornutum* [35], [36]. In order to study *in vivo* the functionality of the DDE in the DD cycle and in NPQ and to refine previous physiological investigations on the relationship between the XC and NPQ in diatoms [18], [19], [24], we targeted the gene encoding the VDE/DDE in *P. tricornutum* [15]. The results suggest successful suppression of the *Vde*/*Dde* gene expression and enabled a comprehensive functional comparison between WT and silenced transformants. To our knowledge this is the first report of genetic manipulation of both the XC and NPQ together in an alga with secondary plastids.

## Results and Discussion

### Silencing the Violaxanthin De-epoxidase (*Vde*) Gene Expression in *P. tricornutum*

**Figure 1:**
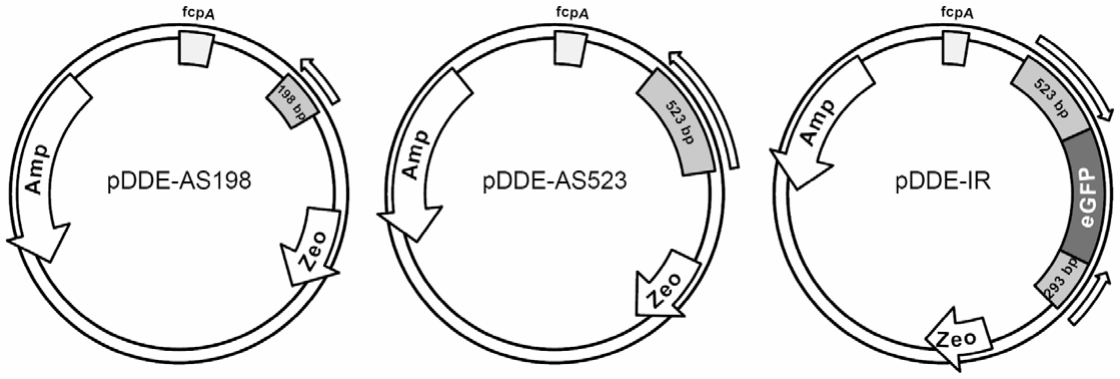
Schematic vector maps of the silencing constructs used for transformation. Anti-sense constructs: *Dde* fragments of 198 or 523 bps were cloned in anti-sense orientation downstream of the *fcpA* promoter (pDDE-AS198 and pDDE-AS523). Inverted repeat constructs: *Dde* fragments of 293 and 523 bp lengths were cloned in sense and anti-sense orientation. The two fragments were linked with an *eGFP* fragment supposed to function as spacer. Amp: Ampicillin resistance; Zeo: Zeocin™ resistance; fcpA: Fucoxanthin Chlorophyll *a*/*c*-binding Protein A promoter; eGFP: enhanced green fluorescent protein.

A *Vde* gene encoding a protein related to the violaxanthin de-epoxidase (VDE) of higher plants (Phatr2 ID 44635) has been identified in the genome of *P. tricornutum* [15], [16]. The deduced protein sequence (ID 44635) shows considerable similarity to the VDE of *Chrysanthemum x morifolium* (61% amino acid sequence identity). Here, it is further referred to as DDE for diadinoxanthin (DD) de-epoxidase and the corresponding gene will be referred to as *Dde*. Two different types of vectors were created, both based on the transformation vector pPha-T1 [37]. The constructs contained either fragments complementary to the partial mRNA sequence (anti-sense, pDDE-AS) of the *Dde* gene or self-complementary inverted repeats (see Fig. 1), which can fold back on themselves to form a dsRNA hairpin-like structure (inverted repeats, pDDE-IR). To investigate whether various lengths of the anti-sense constructs may influence silencing efficiency, we generated two different anti-sense constructs containing complementary *Dde* fragments of 198 and 523 bp length, respectively (pDDE-AS198 and pDDE-AS523). The pDDE-IR construct containing the inverted repeat fragment additionally carries an *eGFP* expression cassette as spacer between the self-complementary components. The intent of this design was to enable visual identification of transformants incapable of RNA interference mediated gene silencing. In such a case stable transcripts would express the fluorescent marker protein. However, we were not able to detect GFP fluorescence in any of the analyzed transformants. DNA containing the gene silencing vectors were introduced into *P. tricornutum* via particle gun bombardment [37], [38] and screened for Zeocin™ resistant colonies.

### Screening of the *Dde* Transformants of *P. tricornutum* using Chlorophyll Fluorescence

**Figure 2:**
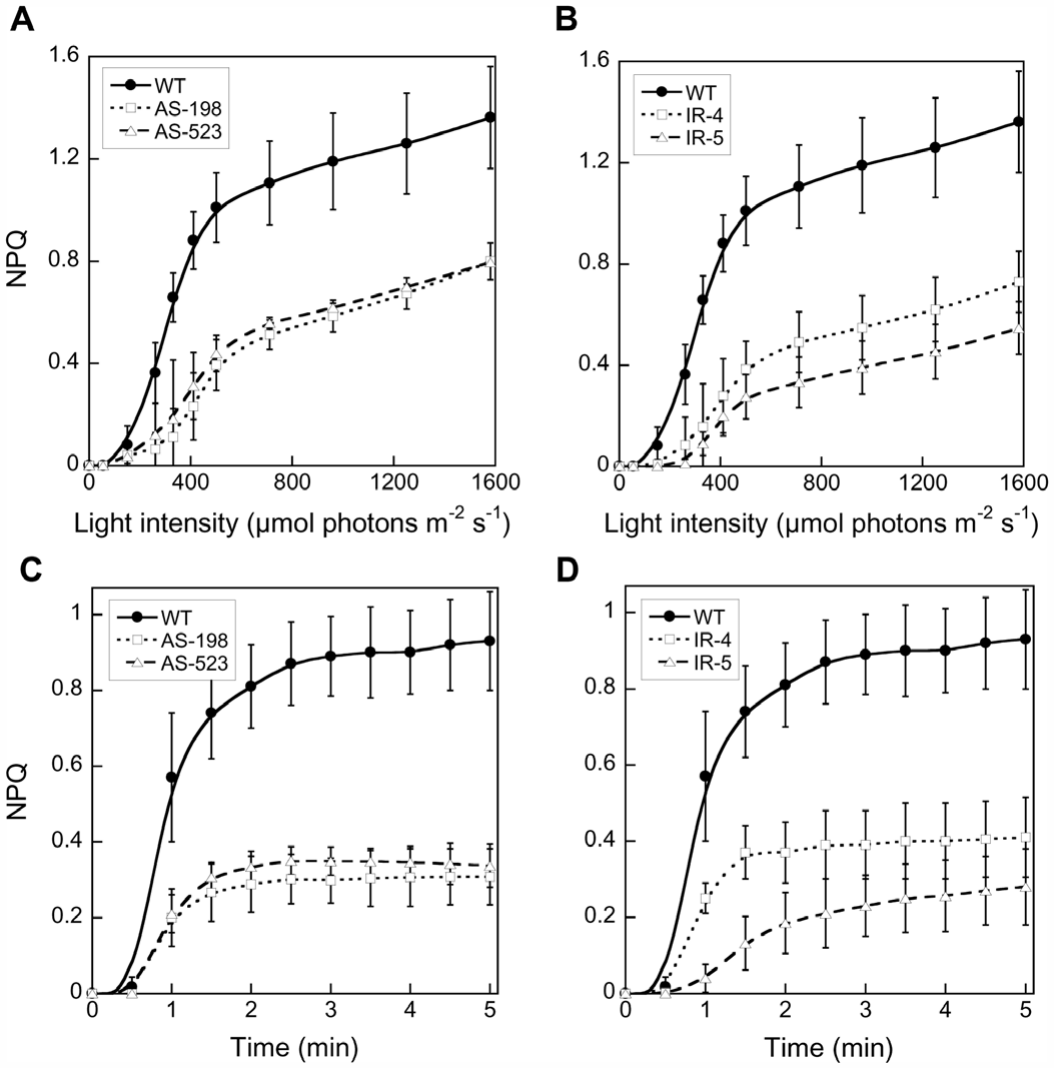
NPQ development in WT and the *Dde* transformants of *Phaeodactylum tricornutum*. Cells were grown at low light (45 µmol photons⋅m^−2^⋅s^−1^) before the experiment. (A) and (B) show the respective NPQs as a function of light intensity for a 5 min light exposure, while in (C) and (D) NPQ was measured as a function of time at an irradiance of 450 µmol photons⋅m^−2^⋅s^−1^. Values are averages ± standard deviation (SD) of three to four measurements.

As the DDE catalyzes the conversion of diadinoxanthin (DD) to diatoxanthin (DT) and as the amount of DT is correlated with the development of non-photochemical fluorescence quenching (NPQ) [2], [9], fluorescence measurements were performed to screen the *Dde* transformants for a possible NPQ phenotype. NPQ manifests itself by a decrease in the quantum yield of chlorophyll fluorescence which allows easy and rapid screening of photosynthetic transformants and mutants, especially in organisms with impaired xanthophyll cycle (XC) and NPQ [39], [40] (see Fig. S1). The measurements revealed that at least 80% of the transformants showed a significantly decreased NPQ (at least more than 10%). The majority of the examined transformants even showed an NPQ reduction between 30% and 47% compared to wildtype cells (WT) (data not shown). *Dde* transformants showing the largest impairment in comparison to the WT strain were selected and NPQ was recorded as a function of light intensity (Fig. 2A and B) and as a function of time at an irradiance of 450 µmol photons⋅m^−2^⋅s^−1^ (intensity for which NPQ reaches saturation in *P. tricornutum* [21], Fig. 2C and D). Both AS and IR *Dde* transformants showed a delayed induction of NPQ and a decreased amplitude as a function of light intensity (Fig. 2A and B). While NPQ started to develop at 150 µmol photons⋅m^−2^⋅s^−1^ in the WT cells, this threshold was raised to up to 250 µmol photons⋅m^−2^⋅s^−1^ in the most impaired transformant (IR-5). In comparison to WT cells the maximal NPQ value was decreased by 43 to 64% (for AS-523 and IR-5) in the *Dde* transformants. When recorded over time (Fig. 2C and D), differences were even more pronounced, especially regarding the amplitude of NPQ (−56% up to −71% for IR-5). In transformant IR-5 also the pattern of NPQ development was different: NPQ induction was much slower during the first 2 min of illumination. Interestingly, in some of the AS transformants, the altered NPQ phenotype disappeared after about 4 months of continuous cultivation, independent of the length of the *Dde* gene used for the transformations (198 bp or 523 bp) (data not shown). In other transformants, the NPQ phenotype remained stable over a period of at least 7 months. In contrast, the NPQ phenotype of the IR transformants was stable over at least two years. While our case indicated that genetic transformation with AS constructs can be transitory and IR transformations are genetically more stable, other reports show no difference in temporal stability after gene silencing with anti-sense or inverted repeat constructs [35].

### Relative Quantification of *Dde* Transcript Levels via Real-time PCR

**Figure 3:**
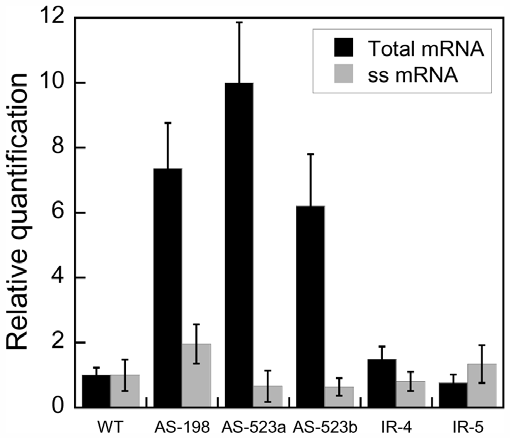
Relative quantification of *Dde* transcripts in the WT and five *Dde* transformants of *Phaeodactylum tricornutum*. Transcript levels are relative to the WT and normalized to *Gapdh* expression. Values are averages of at least two replicates. Black bars: total RNA, untreated RNA used for qPCR; Grey bars: ssRNA, single stranded RNA (total RNA treated with RNaseIII).

The strong NPQ phenotype of the *Dde* transformants suggests successful silencing of *Dde* gene expression. To confirm this finding, the *Dde* transcript levels were determined in six selected AS and IR transformants and were compared to RNA levels of WT cells via quantitative PCR (qPCR). All analyzed AS transformants showed elevated *Dde* transcript levels compared to WT cells (Fig. 3 and table S2). Relative transcript quantities (RQ values) in the transformants AS-523a, AS-523b and AS198 were 6 to 10 fold higher. Interestingly, the *Dde* transcript levels in the IR transformants were similar to that of WT cells. Similar results were reported previously after silencing a phytochrome gene in *P. tricornutum* using inverted repeat constructs. The authors suggest that two different silencing mechanisms might be present that are based on transcriptional and/or mRNA splicing and translation inhibition [35].

To investigate whether the elevated transcript levels in the AS transformants may simply result from accumulation of double-stranded RNA (dsRNA) due to over-expression of the AS construct, we conducted RNase III assays. For that purpose total RNA from WT, AS and IR transformants was treated with RNase III, specifically digesting dsRNA, prior to reverse transcription and qPCR analysis. This treatment led to a significant decrease of *Dde* RQ values for all AS transformants (Fig. 3) revealing approximately equal amounts of single-stranded (ssRNA) *Dde* transcripts in WT and transformant cells. This finding strengthens our hypothesis that the elevated *Dde* transcript levels found in total RNA of AS transformants are indeed the result of an intracellular accumulation of dsRNA. In contrast, RNase III treatment had no significant effect on the relative amounts of *Dde* transcript in the IR transformants. Relative quantities of *Dde* transcript detected for reverse transcribed ssRNA (RNase III treated) were not significantly different to those observed in reverse transcribed total RNA (untreated) of IR-4 and IR-5.

Attempts to measure the DDE protein level of WT cells and the *Dde* transformants by Western Blot analysis with a heterologous antiserum raised against VDE from lettuce (kindly provided by Prof. Harry Y. Yamamoto, University of Hawaii) unfortunately failed. Also, several approaches to generate antisera against *P. tricornutum* DDE remained unsuccessful. However, it has been shown in *P. tricornutum* [35], but also in *Caenorhabditis elegans* [41], that in silenced transformants with unchanged levels of mRNA, the respective protein levels can be reduced. In plants, endogenous micro RNAs (miRNA) may induce silencing of their target RNA by translational repression. While transcript levels of the target RNA were unaffected, protein levels differed between WT samples and samples over-expressing the miRNA [42], [43].

### Pigment Content, Photosynthetic Properties and Growth Rate of the WT and the *Dde* Transformants of *P. tricornutum*

To find out how silencing of *Dde* potentially affects the physiological properties of *P. tricornutum*, pigment content, photosynthetic properties and growth were evaluated in WT cells and the *Dde* transformants. The chlorophyll *a* (Chl *a*) content per cell and the amount of light-harvesting pigments (Chl *c* and fucoxanthin) and β-carotene were similar in WT and *Dde* transformants (+/− 7–9%) (table 1). As β-carotene is mainly found in the reaction center of the photosystems [44], it is a good indicator for the number of photosystems per Chl *a*. Accordingly the β-carotene content was similar in the WT cells and the *Dde* transformants. The amount of diadinoxanthin (DD) also was not significantly different in WT cells and the AS and IR transformants (student test, p<0.05). At the chosen light conditions (45 µmol photons⋅m^−2^⋅s^−1^, 16∶8 h light:dark), no diatoxanthin (DT) was detectable as previously reported [22], [44]. There was also no significant difference in the maximum photosynthetic efficiency of PSII (F_v_/F_m_) and in the maximal relative rate of linear electron transport (rETR_max_) between the WT and the *Dde* transformants (table 1). The maximal light use efficiency (α) was similar except for IR-4 in which it was slightly reduced as illustrated by the lower content of Chl *a* per cell. The light intensity needed to reach rETR_max_ (E_m_) was similar for all types of cells. These data indicate that the photosynthetic ability of the WT cells and *Dde* transformants grown under low light was generally comparable. Finally, the growth rates of WT cells and of the *Dde* transformants were also similar. Hence, it is reasonable to argue that the expression of the constructs did not disturb the biosynthetic pathway of the pigments including the xanthophylls, neither the ability of the photosynthetic apparatus to perform photochemistry nor the cells’ ability to produce biomass under low light conditions.

**Table 1:** Pigments and photosynthetic properties of WT and the *Dde* transformants of *Phaeodactylum tricornutum.*

	WT	AS-198	AS-523	IR-4	IR-5
**Chl ** ***a***	0.27±0.01	0.29±0.01	0.25±0.01	0.25±0.02	0.27±0.01
**Chl ** ***c***	14.0±0.5	13.8±1.2	13.6±0.6	14.5±0.8	13.7±0.6
**Fucoxanthin**	68.3±4.1	67.8±5.0	66.5±3.5	71.5±5.8	65.3±6.4
**DD**	8.6±1.0	7.6±1.4	7.1±0.7	6.3±1.6	6.3±1.5
**ß-carotene**	7.0±2.1	6.8±2.3	8.3±3.6	7.2±3.7	7.1±3.0
**F_v_/F_m_**	0.71±0.02	0.72±0.06	0.70±0.03	0.71±0.05	0.73±0.05
**rETR_max_**	41.6±1.7	43.9±1.7	44.5±1.5	38.8±2.4	39.9±1.4
**α**	0.20±0.02	0.19±0.02	0.18±0.07	0.16±0.05	0.19±0.05
**E_m_**	453±12	441±3	420±12	422±8	461±14
**μ**	1.00±0.06	1.04±0.02	1.01±0.12	0.93±0.02	0.97±0.07

Pigment content (in mol/100 mol Chl *a*) and photosynthetic properties of the WT and the *Dde* transformants of *P. tricornutum* cells grown under low light (45 µmol photons⋅m^−2^⋅s^−1^). Chl *a* is given in pg cell^−1^, DD: diadinoxanthin, F_v_/F_m_: the maximum photosynthetic efficiency of photosystem (PS) II, rETR_max_: is the relative maximal rate of linear electron transport (µmol photons⋅m^−2^⋅s^−1^), α: the maximum light used efficiency (in µmol photons⋅m^−2^⋅s^−1^), E_m_: the light intensity needed to reach rETR_max_ in µmol photons⋅m^−2^⋅s^−1^, μ: the growth rate in d^−1^. Values are averages, ± SD of seven to nine measurements for the pigment data (except Chl *a*, three measurements) and three to four measurements for the other data.

### Diadinoxanthin (DD) De-epoxidation in the WT and *Dde* Transformants of *P. tricornutum* Grown Under Low Light (LL)

To investigate the relevance of *Dde* for the de-epoxidation of DD to DT we compared the de-epoxidation states (DES) between WT and silenced transformants by quantifying the amounts of DD and DT after a short illumination (5 min) of moderate high light (450 µmol photons⋅m^−2^⋅s^−1^). The selected irradiance was the light intensity needed to reach the maximal rETR (439±18 µmol photons⋅m^−2^⋅s^−1^) in all types of cells. It was shown before that this intensity is sufficient to generate a steady-state in DT synthesis [18]. Compared to WT cells, DES was on average 50% lower in the transformants: 29.5±4.5% and 16.4±1.3%, respectively (table 2). Also, the amount of DT synthesized after 5 min illumination was on average 62% lower in transformants (1±0.1 mol/100 mol Chl *a*) compared to the WT (2.6±0.4 mol/100 mol Chl *a*) (table 2). It is known that in diatoms a lower pool size of DD results in a lower degree of de-epoxidation and DT content [21]. This apparently could be partially the case in the IR transformants which showed a slightly lower DD pool size (table 2). However, as the DD pool size of the AS transformants was not lower than in WT cells (table 1), we believe that the main reason for a lower DES and DT amount in the transformants was not due to a reduced DD substrate availability, but rather to reduced amounts of DDE enzyme, which could not be verified because of the lack of a reliable DDE antibody. By addition of 20 µM dithiothreitol (DTT), a well-known inhibitor of the DDE [18], we were able to artificially reproduce the decrease of DD de-epoxidation and DT amounts in the WT cells by partially inhibiting (by about 50% at this concentration, according to [18]) the activity of the DDE (table 2). Two other factors that can have direct effects on the DES [9] are: i) the availability of the acidic form of ascorbate (an essential co-factor of the DDE) [19], [45] and ii) the ability to build up a transthylakoidal ΔpH, which is essential to activate the enzyme [9], [18], [24], [45]. It is rather unlikely that those two factors are the reason for the lower steady-state DES, regarding the unaffected ability of the transformants to perform photosynthesis and to grow. Interestingly, both silencing approaches, AS and IR, were similarly efficient with up to 50% suppression of DD de-epoxidation ability. This was drastically less than the 95% suppression reported for the silencing of *Vde* in higher plants [12]. Furthermore, as all AS transformants showed the same impairment in DD de-epoxidation (−54.9±4.4%), we conclude that the length of the partial *Dde* sequence did not play a role in the silencing efficiency.

**Table 2:** Diadinoxanthin and diatoxanthin (DD and DT) content and de-epoxidation state (DES) of the WT and *Dde* transformants of *Phaeodactylum tricornutum* grown under low light (LL).

	WT	WT+DTT	AS-198	AS-523	IR-4	IR-5
**DD**	6.3±0.8	8.2±0.4	6.2±1.3	5.6±0.8	5.2±0.5	4.7±0.5
**DT**	2.6±0.4	1.1±0.1	1.1±0.2	1.1±0.1	1.1±0.1	0.9±0.2
**DD+DT**	8.9±0.9	9.3±0.4	7.3±1.5	6.8±0.8	6.3±0.4	5.9±0.7
**DES**	29.5±4.5	12.2±1.4	14.9±0.9	17.5±2.5	18±1.3	15.25±2.3

Diadinoxanthin (DD) and diatoxanthin (DT) content (in mol/100 mol Chl *a*), and the de-epoxidation state (DES) of the WT (+/− dithiothreitol, DTT) and the *Dde* transformants of *P. tricornutum* cells (LL grown) after a 5 min 450 µmol photons⋅m^−2^⋅s^−1^ light treatment. DES (in%)  =  DT/(DD+DT) × 100. Values are averages ± SD of three to five measurements.

**Figure 4:**
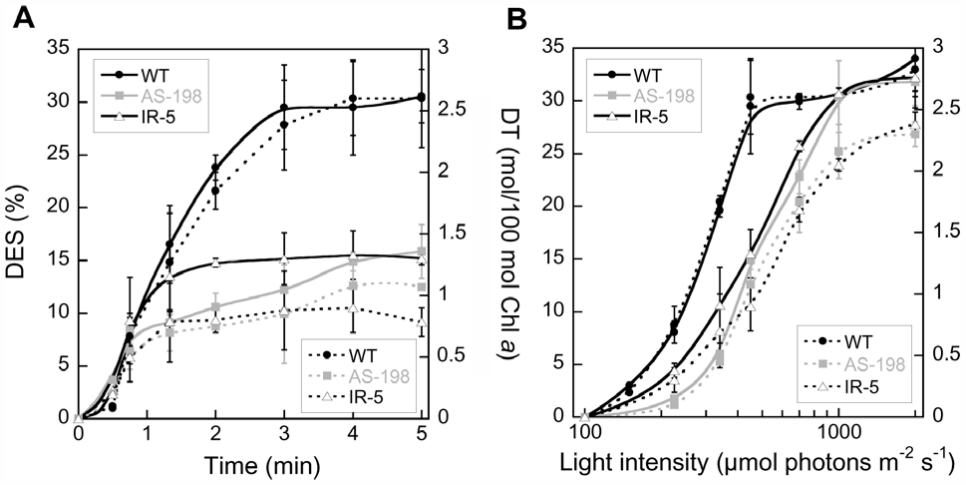
De-epoxidation state (DES) and diatoxanthin synthesis in the WT and two *Dde* transformants of *Phaeodactylum tricornutum* cells grown under low light as a function of time at an irradiance of 450 µmol photons⋅m^−2^⋅s^−1^ (A) and as a function of light intensity for a 5 min light exposure (B). A new sample was used for each time and for each irradiance. Values are averages ± SD of three to four measurements.

To gain more insight into the DD de-epoxidation and DT amount, we measured these two parameters in WT, AS-198 and IR-5 cells at the same two light exposure conditions used for measuring NPQ (Fig. 2). The DD de-epoxidation time kinetics showed that the DT synthesis already started after 30 s, and after 1.5–2 min of illumination we could detect a clear difference between the WT and the *Dde* transformants (Fig. 4A). DD de-epoxidation in the WT cells and AS-198 reached a plateau after 3 to 4 min, while saturation was reached earlier in IR-5 (after 2 min). Maximal DES was much lower in these transformants. After progressively increasing the light intensity from darkness up to 2000 µmol photons⋅m^−2^⋅s^−1^ (full sunlight in nature, [46]), the pattern of DD de-epoxidation rates observed in the WT and the *Dde* transformants were different (Fig. 4B). No de-epoxidation was observed below 150 µmol photons⋅m^−2^⋅s^−1^ (as previously reported, [21], [27]) for all types of cells. In WT cells, DD de-epoxidation reached a plateau at much lower light intensities than the *Dde* transformants (around 500 µmol photons⋅m^−2^⋅s^−1^ and 1000 µmol photons⋅m^−2^⋅s^−1^, respectively) and the efficiency of DD de-epoxidation before reaching saturation (slope of the linear phase in Fig. 4B) was two times higher in WT (0.095% photons^−1^) compared to the *Dde* transformants (0.044% photons^−1^ and 0.042% photons^−1^ in AS-198 and IR-5, respectively). Still, the DES reached for over-saturating intensities was the same in WT and *Dde* transformants (30–35% from around 1000 µmol photons⋅m^−2^⋅s^−1^), even though the amounts of DT synthesized remained slightly higher in the WT (by 15–20%).

The activity of DDE is regulated by the transthylakoidal ΔpH [9], [17], [24], [45], [47] which itself depends on the extent of ETR. As a consequence, the activity of the global pool of DDE enzymes responsible for the de-epoxidation of DD is likely to be indirectly regulated by ETR via the coupled generation of the transthylakoidal ΔpH. We indeed recently observed that a decreased ETR can indirectly alter the DD de-epoxidation in *P. tricornutum* ([48] and unpublished data). The activity of DDE pool therefore progressively increased with the light intensity until a maximum already before the saturation of photochemistry at E_m_ was reached as observed for WT cells (Fig. 4B). At E_m_ and above, the DD de-epoxidation drastically slowed down most probably because the DDE enzymes were product-limited in two ways. First, there is a part of the DD pool which cannot be de-epoxidized because it is not accessible to the DDE [2]. Although the fraction of this pool depends on the species [22], [23], [49], the irradiance/light climate the cells were acclimated to [25], [44], [50]–[53] as well as the light treatment [22], [23], [54], it is often reported to reach about 40–50% under similar light conditions [2]. Secondly, it was shown in higher plants that the VDE can be inhibited by the binding of its pigment product, zeaxanthin (Zx), to the active sites of the enzyme [55]. This feed-back inhibition is strong and fast enough to compete with the de-epoxidation. It is thought to support the fine regulation of the de-epoxidation as a function of the Zx amount, especially when Zx becomes excessive. Regarding the similarities of the VDE and the DDE characteristics [9], [15], it is reasonable to extrapolate this feature for the DDE and DT.

In the *Dde* transformants, the correlation between the light intensity and the DD de-epoxidation was disturbed: it was shifted towards higher intensities up to a threshold which was about two times E_m_. As argued above, there is no reason to suspect ETR and the subsequent build-up of the transthylakoidal ΔpH to be different in WT cells and *Dde* transformants. Nevertheless, the demonstration that the addition of DTT in WT cells generated the same low DES as in the *Dde* transformants supports the disturbance of the control of the activity of the DDE enzymes by the acidification of the thylakoid lumen [9], [18]. We propose that because of the lower DDE concentration, the progressive activation of the global pool of DDE enzymes as a function of the lumen acidification and the irradiance was slower, which means that the same lumen acidification activates fewer DDE enzymes. This was clearly illustrated by the lag phase of DD de-epoxidation at the lowest light intensities as well as the lower efficiency for higher irradiances up to 1000 µmol photons⋅m^−2^⋅s^−1^ (Fig. 4B). This was supported by the lower DES in the *Dde* transformants already after 45 s-1 min illumination (Fig. 4A). The observation that DES was similar in the *Dde* transformants and WT cells before 45 s-1 min at E_m_ probably illustrates the fact that the DDE concentration in the *Dde* transformants was sufficiently high to ensure a basal level of DES under the conditions of low transthylakoidal ΔpH as shown before [17], [18] (see also below). This feature could be amplified by the activity of the epoxidase enzyme which catalyzes the back conversion of DT into DD. As its constant rate is comparable to the one of DD de-epoxidation [9], DT epoxidation competes with DD de-epoxidation up to a certain high light/strong transthylakoidal ΔpH threshold where it is inhibited [24], [56]. Under these conditions, and as observed here for WT cells, maximal DD de-epoxidation occurred (Fig. 4B). In the *Dde* transformants, because of the low amount of DDE, the activity of the DT epoxidase enzymes could out-compete the DDE activity at low/moderate intensities. Interestingly, for intensities above 1000 µmol photons⋅m^−2^⋅s^−1^, the DES in the *Dde* transformants was similar to the one of WT cells. This can be explained by the inhibition of the DT epoxidase at these high intensities (from 800 µmol photons⋅m^−2^⋅s^−1^ on in [24]) which stopped the back-conversion of DT and allowed a maximal accumulation of DT in the *Dde* transformants albeit the lower DDE concentration. The ability of the *Dde* transformants to reach the same DES than WT cells at maximum irradiance further supported the assumption that de-epoxidation was not ascorbate-limited. Additionally, it is possible that the disturbance of the balance between the control of the DDE enzymes by the lumen acidification and their feed-back inhibition by DT would be less significant for high irradiances/low luminal pH, albeit the higher DT concentration.

A change in the accessibility of the DD molecules to the DDE related to a different localization of DD [2], [54], [57] and/or to a different lipid composition [4], [9], [58] in the *Dde* transformants can be ruled out as the kinetics/light conditions needed for these processes [25], [44] do not fit with our experimental conditions.

### NPQ Development *versus* the Synthesis of Diatoxanthin (DT) in the WT and *Dde* Transformants of *P. tricornutum* Grown Under LL

**Figure 5:**
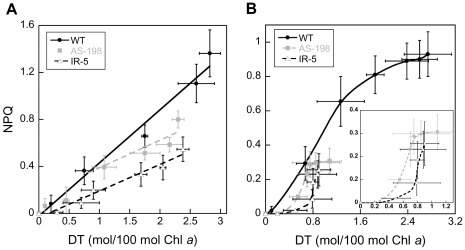
NPQ development *versus* diatoxanthin (DT) amount in WT and two *Dde* transformants of *Phaeodactylum tricornutum* cells grown under low light. (A): NPQ and DT data obtained as a function of light intensity for a 5 min light exposure (data from Fig. 2A and 2B, and Fig. 4B), (B): NPQ and DT were measured as a function of time at an irradiance of 450 µmol photons⋅m^−2^⋅s^−1^ (data from Fig. 2C and 2D, and Fig. 4A). The linear NPQ/DT relationships in (A) obtained for WT was NPQ  = 0.45 DT (R^2^ = 0.98), for AS-198 NPQ  = 0.24 DT (R^2^ = 0.83) from about 1 mol DT/100 mol Chl *a*, and for IR-5 NPQ  = 0.23 DT (R^2^ = 0.97). Values are averages, ± SD of three to four measurements. See the text for details.

The relationship between the synthesis of DT and the development of NPQ shows a linear relationship [2], [21], [22], [24], [25], illustrating the tight link between NPQ and DT in diatoms [2], [4], [9]. In WT cells, such a relationship with a similar slope (0.45) could be observed when the cells were exposed to increasing light intensities (Fig. 5A) and illumination times (Fig. 5B, first part of the relationship up to about 1.3 DT mol/100 mol Chl *a*). The slope of the relationship was 50% lower than the one previously reported for the same species [21], [22], stressing the possibility of different NPQ abilities in individual strains of *P. tricornutum* [36]. In the *Dde* transformants the NPQ/DT slope was lower than the one of the WT cells: from about 1 and 0 mol DT/100 Chl *a* on for AS-198 and IR-5, respectively (Fig. 5A). This indicates that at the same irradiance the *Dde* transformants synthesized less DT (Fig. 4B) but additionally, that there were less DT molecules involved in NPQ and/or that their quenching efficiency was lower (up to 50% less in the case of IR-5; NPQ/DT slope  = 0.23). A lower involvement/efficiency of DT in NPQ has been reported before for *P. tricornutum* [25], which was due to a change of balance between different DD/DT pools showing spatial and functional heterogeneity [25], [54]. DD/DT molecules are distributed among several pools: free in the lipid matrix of the thylakoid membranes, lipid diluted in the vicinity of the LHC antenna of photosystems and protein-bound to the specific PS I LHC antenna and bulk peripheral LHC antenna [54], [57]. While only the last pool is known to effectively participate in NPQ, the lipid-localized DT molecules help to prevent lipid peroxidation and may also serve as a reservoir for fucoxanthin synthesis [2], [9], [53], [54]. Although it is likely that VDLs and VDRs might be de-epoxidases based on their high homology with VDE/DDE [15], [16], so far, it remains unknown which of them might be responsible for the de-epoxidation of which of the DD pools [15]. VDL and VDR enzymes might have a different localization and functional role than DDE as indicated by the differential light-induced expression of the *Vdl* genes compared to the *Dde* (or *Vde*) gene [15] and by their putative lower ability to be regulated by protonation as suggested by their differential amino acid content [15], [16]. Therefore, it must be assumed that while the activity of VDL and VDR enzymes might still be controlled by protonation as DDE [8], [11]–[13], they most probably have a different/lower reactivity to pH changes. As a consequence, the VDLs and VDRs probably lack an essential feature for their involvement in the XC, at least the way DDE is involved in the XC, which is the fine regulation of their activity as a function of the lumen acidification. It is thus probable that either i) they are localized in the lumen of thylakoids but their activity is differently regulated as a function of the light-dependent build-up of the transthylakoidal ΔpH as already reported for de-epoxidases from different organisms [17], [47], and/or ii) they are not localized in the lumen of the thylakoids but in the lipid matrix of membranes, as proposed for one of the DT epoxidases [59]. Regarding the fact that VDLs but not VDRs [15] have a slightly charged C-terminus, it is also possible that VDLs would be part of the first group and VDRs of the second. All alternatives do not interfere with the recent model of the organization of thylakoid membranes in diatoms [4].

In any way, the first case would lead to a decoupling between irradiance, ΔpH, DD de-epoxidation and NPQ as reported earlier [18], [19] while the second case would lead to the accumulation of DT molecules in the lipids where they have a higher probability to be involved in the prevention of lipid peroxidation [11], [54] instead of participating in NPQ [22], [25]. In both cases, and only if the amount of DT synthesized by VDLs and/or VDRs would be significant relative to the DT molecules which participate to NPQ, it would generate a decrease of NPQ for a given amount of DT and irradiance (i.e. a decrease of the slope of the NPQ/DT relationship). Such a situation was previously reported in high light acclimated diatom cells in which the amount of DT in the lipids was higher [25], [54]. This is fitting well to the increase of the transcript level of *Vdl2* but also of *Vdr1* and *Vdr2* genes under high light exposure [5]. It is unlikely that DT *de novo* synthesis would explain the NPQ/DT slope difference in the *Dde* transformants [22], [60] because of the 5 min light treatment and because it may be assumed that *de novo* DT synthesis would occur at any irradiance.

Because of the decreased DT amounts synthesized by DDE at all irradiances (Fig. 4B) in the *Dde* transformants, we propose that the amount of the DT molecules synthesized by the VDL and/or VDR enzymes and not involved in NPQ increased relative to the DT molecules involved in NPQ. As a consequence, the slope of the NPQ/DT relationship was lower for all irradiances (Fig. 5A). It additionally brings an alternative explanation for a part of the DES *versus* irradiance pattern (Fig. 4B). Nevertheless, while it was true for IR-5, this conclusion is true for AS-198 only for intensities above E_m_ (Fig. 5A). Above this intensity, which corresponds to a synthesis of about 1 mol DT/100 mol Chl *a*, the slope of the NPQ/DT relationship changed from 0.45 (as the WT) to 0.24 (as IR-5). This pattern means that below E_m_, most of the DT molecules participated to NPQ while above E_m_, DT molecules not involved in NPQ started to be significant. Such a conclusion implies two assumptions: i) in AS-198 the DDE concentration was higher, and/or the VDL and/or VDR concentrations were lower; an assumption which was not possible to test without any reliable antibody, ii) the global pool of VDL and VDR enzymes needs a certain threshold of light and lumen acidification to be fully active, thus fitting with their lower/different pH sensitivity.

The explanation above was further supported by the pattern of the NPQ/DT relationship when the cells were exposed to E_m_ for increasing times (Fig. 5B). In WT cells, the slope of the NPQ/DT relationship decreased from 0.44 to 0.13. It likely illustrated a change of balance from a dominant synthesis of DT molecules bound to the LHC proteins to a higher synthesis of DT molecules localized in the lipids. Following the hypothesis that the former would be mostly generated by DDE and the latter mostly by the VDLs and/or VDRs, it would support the second assumption above, i.e. VDLs and VDRs need a stronger lumen acidification (and hence a longer illumination time) as well as a higher intensity to be fully active (AS-198, Fig. 5A). In the *Dde* transformants, we observed a phase at the beginning of the illumination with synthesis of DT without any NPQ onset (Fig. 5B). This continued until a DT concentration of about 0.2 and 0.4 mol/100 mol Chl *a* was reached in AS-198 and IR-5, respectively (insert of Fig. 5B), corresponding to about 30–45 s illumination (Fig. 4A). Afterwards the NPQ developed in parallel with the DT amount in an exponential/linear rise until it slowed down and stopped as observed for WT cells but at lower values and DT amounts as expected. This last phase was reached after about 1.5 min of illumination which was just before the DD de-epoxidation reached a plateau (Fig. 4A).

Such discrepancies between DT synthesis and NPQ were documented in *P. tricornutum* by artificially manipulating the build-up of the transthylakoidal ΔpH [18], [19]: a low ΔpH can generate a significant amount of DT with nearly no NPQ. This is the result of the high sensitivity of DDE to a weak lumen acidification [47], [17], while the acidification is not strong enough to generate the so-called ‘activation’ of DT molecules in order to trigger NPQ [19], [24]. Such ‘activation’ is likely to occur via the protonation of some PS II LHC binding sites during the acidification of the lumen [19], [20]. Two reasons could thus explain the delay of the onset of NPQ *versus* DT in the *Dde* transformants at the beginning of the illumination, as well as for low irradiances especially in IR-5 (Fig. 5A, DT concentration below 0.5 mol DT/100 mol Chl *a*): i) a fewer number of proton binding sites of the PS II LHC proteins, ii) a lower protonation of these sites, their number being stable. The second option is unlikely because, as described above, it necessitates a lower translocation of protons coupled with the photosynthetic transport rate of electrons in the *Dde* transformants which was not the case (table 1). The first option, although plausible, is impossible to verify: at the present state of knowledge, we do not know which of the LHC proteins might bind protons in diatoms. Nevertheless, LHCx proteins have been recently proposed to potentially play such a role [4], [36]. If the *Dde* transformants would synthesize less LHCx proteins, this would imply a co-regulation of the transcription of *Lhcx* and *Vde*/*Dde* genes and/or of the synthesis of LHCx and VDE/DDE. After a certain lumen acidification was reached, i.e. for longer illumination, an exponentially increasing amount of DT molecules became activated and NPQ strongly developed until slowing down for the reasons described above. In WT cells, there is no uncoupling between lumen acidification, DT synthesis, DT activation and NPQ as usually reported [18], [19], [24] and as observed here. It thus further supports our assumption of an uncoupling in AS-198 and IR-5 probably because of a simultaneous differential activation of i) the pool of DDE enzymes because its concentration was lower and of ii) the pool of VDL and/or VDR enzymes because of their putative different/lower pH sensitivity. As NPQ needed more DT to be triggered and to be further developed in IR-5 than in AS-198 transformants, it has to be assumed that either the DDE concentration was lower, and/or VDL and/or VDR concentrations were higher in IR-5. It supports the observed differences of NPQ/DT relationship for low irradiances between IR-5 and AS-198 (Fig. 5A). It is possible that the different silencing approach induced an up-regulation of *Vdl* and/or *Vdr* genes and an increased amount of the corresponding enzymes in IR transformants as part of a compensatory reaction to the lower DDE concentration.

### DD De-epoxidation in the WT and *Dde* Transformants of *P. tricornutum* Grown Under ‘High Light’ (HL)

To test whether the silencing effects can be increased without otherwise stressing the cells, we took advantage of the light dependent *FcpA* promoter which was driving the transcription of the silencing constructs. We performed the same experiments as before, but cultivated *P. tricornutum* at 3 times higher light intensities (135 µmol photons⋅m^−2^⋅s^−1^). A similar irradiance, which is close to E_k_ in *P. tricornutum* and other diatoms [27], [48], [61], was used previously to grow *P. tricornutum* in a number of photo-physiological studies [50], [60], [62], [63]. At these growth conditions, F_v_/F_m_ was similar in the WT cells while the growth rate slightly increased (16%) compared to LL conditions (table 3). F_v_/F_m_ in the *Dde* transformants was very similar to that of WT cells, while their growth rate was lower (−12%) than in the WT cells but remained stable or slightly increased compared to LL conditions (tables 2 and 3). These results indicate that both WT and *Dde* transformant cells were not stressed. The pigment content of WT and the *Dde* transformants remained mostly identical, with the exception of the amounts of DD and DT (table 3). The DD pool size was increased in all cells, as generally reported for *P. tricornutum* grown under HL [25], [49]. Such an increase was shown to enhance the ability of the cells to produce DT [44], [51], [52] thus elevating the photoprotection efficiency, especially by increasing the amplitude of NPQ [4], [20], [21], [25], [49]. Interestingly, while the amount of DD+DT increased by a factor of 1.7±0.1 in the WT and AS transformants, it was higher by a factor of 2.1 in the IR transformants. As a consequence and in comparison to the LL growing conditions (see table 1), the pool size of DD was the same in all the cells (13.5±0.6 mol/100 mol Chl *a*). Additionally and in contrast to LL growing conditions, DT molecules (0.68±0.12 mol/100 mol Chl *a*) ‘constitutively’ accumulated due to the higher light excitation pressure (as reported before [4], [49], [60]) confirming that DD de-epoxidation starts between 100 and 150 µmol photons⋅m^−2^⋅s^−1^ (Fig. 4B).

**Table 3:** F_v_/F_m_, growth rate, diadinoxanthin (DD) and diatoxanthin (DT) content and de-epoxidation state (DES) of the WT and *Dde* transformants *Phaeodactylum tricornutum* grown under high light (HL).

		WT	AS-198	AS-523	IR-4	IR-5
	F_v_/F_m_	0.67±0.02	0.64±0.03	0.63±0.03	0.65±0.04	0.64±0.02
	μ	1.16±0.04	1.03±0.03	1.00±0.08	1.03±0.04	1.06±0.07
Before LT						
	DD	13.0±2.2	13.4±2.2	12.1±3.2	12.4±0.3	12.9±0.6
	DT	0.7±0.2	0.8±0.1	0.8±0.1	0.6±0.2	0.5±0.2
	DD+DT	13.9±2.3	14.2±2.1	12.8±3.2	13.2±0.2	13.6±0.6
	DES	4.8±0.3	5.6±1.0	5.2±2.0	5.3±0.2	4.3±0.6
After LT						
	DD	8.6±1.4	10.2±0.8	8.9±0.8	10.1±0.6	10.4±0.3
	DT	4.9±0.5	2.1±0.3	2.2±0.3	3.0±0.3	2.8±0.1
	DD+DT	13.6±1.9	12.7±0.3	11.9±0.8	13.3±0.1	13.5±0.5
	DES	32.8±2.4	17.0±2.8	18.8±2.7	22.0±2.8	22.0±1.8

Maximum photosynthetic efficiency of PSII (F_v_/F_m_), growth rate (μ in d^−1^), diadinoxanthin (DD) and diatoxanthin (DT) content (in mol/100 mol Chl *a*), and de-epoxidation state (DES) of the WT and the *Dde* transformants of *P. tricornutum* cells grown at HL (135 µmol photons⋅m^−2^⋅s^−1^). DD, DT and DES were determined before and after a 5 min 450 µmol photons⋅m^−2^⋅s^−1^ light treatment (LT). DES (in%)  =  DT/(DD+DT) ×100. Values are averages, ± SD of three measurements.

**Figure 6:**
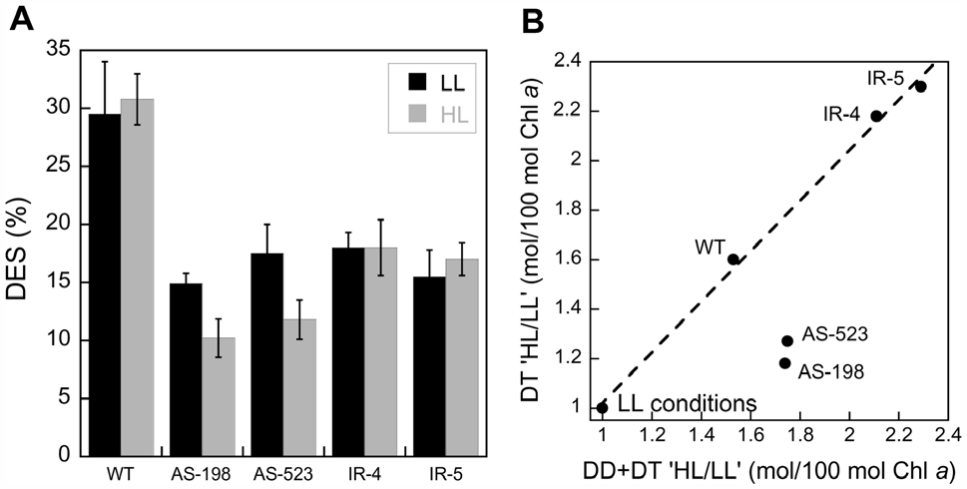
De-epoxidation state (DES) and the relationship between the increase of the diadinoxanthin+diatoxanthin (DD+DT) and the diatoxanthin (DT) amounts in WT cells and the *Dde* transformants of *Phaeodactylum tricornutum*. (A): De-epoxidation state (DES) of the WT and the *Dde* transformants of *P. tricornutum* cells grown under low (LL) and ‘high’ (HL) light (45 and 135 µmol photons⋅m^−2^⋅s^−1^, black and white column, respectively) after a 5 min 450 µmol photons⋅m^−2^⋅s^−1^ light treatment. For the HL cells, the DES obtained during the 450 µmol photons⋅m^−2^⋅s^−1^ light treatment was calculated as the total DES (table 3, data ‘After LT’) minus the DES which already occurred before the light treatment during the HL acclimation (table 3, data ‘Before LT’). Such a calculation was not necessary for the LL acclimated cells as no DD de-epoxidation occurred before the light treatment (see table 1, no presence of DT as this low intensity). Values are average, ± SD of three to four measurements. (B): Relationship between the increase in the total DD+DT pool size and the DT amount synthesized during the light treatment in HL grown cells compared to LL grown cells. For the HL cells, the DT amount synthesized during the 450 µmol photons⋅m^−2^⋅s^−1^ light treatment was calculated as the total DT amount (table 3, data ‘After LT’) minus the DT amount which already occurred before the light treatment during the HL acclimation (table 3, data ‘Before LT’). Thus, the x-axis refers to the increase of the DD+DT pool size under HL acclimation versus the LL conditions while the y-axis refers to the increase of the DT synthesis of cells acclimated to HL versus the DT synthesis of cells acclimated to LL. As a consequence, ‘LL conditions’ refers to the value of the relationship for all type of cells and is equal to 1. The linear relationship obtained for the WT and the IR transformants was DT  = 1.02 (DD+DT) with R  = 0.99. All data were extracted from tables 1, 2 and 3. See the text for details.

When the cells subsequently were exposed to E_m_ (450 µmol photons⋅m^−2^⋅s^−1^) for 5 min, WT cells and the IR transformants showed a similar DD de-epoxidation as under LL conditions during the light treatment. In AS transformants DD de-epoxidation was reduced by 30–35% (Fig. 6A). DD de-epoxidation during light treatment was calculated as the total DD de-epoxidation (table 3, data ‘After LT’) minus the DD de-epoxidation which already occurred during the HL acclimation (table 3, data ‘Before LT’). Such calculation was possible due to the fact that the DT synthesized either during the HL acclimation or during the light treatment was most probably exclusively generated by DD de-epoxidation and not by *de novo* synthesis since the irradiance for the HL acclimation was too low (135 µmol photons⋅m^−2^⋅s^−1^) and the illumination time for the light treatment was too short (5 min) [22]. The same calculation was not necessary for the LL acclimated cells as no DD de-epoxidation occurred before the light treatment because the light intensity was too low for triggering DD de-epoxidation (see table 1) [21], [44]. The amount of DT synthesized by de-epoxidation from DD during the 450 µmol photons⋅m^−2^⋅s^−1^ light treatment (i.e. excluding the amount of DT synthesized during the HL acclimation which equals the DT amount synthesized ‘After LT’ minus the DT amount synthesized ‘Before LT’, table 3) was 61% higher in the WT in comparison to cells at LL (an average of 4.2 and 2.6 DT molecules, respectively), which was similar to the increase (+56%) in the total DD+DT pool size (compare tables 1, 2 and 3). As explained above, this feature is typical when the cells are shifted from LL to higher light conditions: more DD is synthesized so that the subsequent increase in DT is due to an increase in the substrate quantity but not in the quantity and/or the activity of the DDE [21], [25], [44], [51], [52]. This increase might also refer to the up-regulation of the *Dde/Vde* gene as well as *Vdl2* and *Vdr1/2* under HL (although 135 µmol photons⋅m^−2^⋅s^−1^ might be too low [5]) which might lead to an increase in the quantity of the enzymes able to synthesize DT. Based on the light-dependent physiological properties of the DD/DT pool in diatoms [4], we compared the amount of DT synthesized by de-epoxidation from DD exclusively during the 450 µmol photons⋅m^−2^⋅s^−1^ light treatment with the size of the total DD+DT pool under both LL and HL conditions (before the light treatment) for WT cells and the *Dde* transformants (Fig. 6B). In such a plot, WT cells showed an identical ratio of DT versus DD+DT increase from LL to HL conditions (1.61 *versus* 1.56) as expected from the comparison of data in tables 1, 2 and 3. For the IR transformants the same observation was made even though the increase in both DD+DT and DT was higher: ×2.11 and ×2.18 respectively for IR-4, and ×2.29 and ×2.30 respectively for IR-5 (table 3 and Fig. 6B). This increase which appeared higher than in WT cells (about ×1.6) might be only the result of slightly lower DT and DD+DT amounts in LL cells in the IR transformants (table 2) and might not have any physiological importance. Albeit the higher increase of total DD/DT in IR-transformants compared to WT cells, the amount of DT synthesized by de-epoxidation from DD during the 450 µmol photons⋅m^−2^⋅s^−1^ light treatment was about two times lower than in WT cells similar as for the *Dde* transformants and WT cells grown under LL (table 2). This illustrated the stable inability of the transformants to perform DD de-epoxidation independently of the growth light conditions (LL or HL) the cells were acclimated. Surprisingly a different relationship was found in the AS transformants (Fig. 6B). While the size of the DD+DT pool was increased by 74.5±0.5% in AS-198 and AS-523, the corresponding increase in DT synthesized during the light treatment was only 18% and 27%, respectively. Consequently, in AS-198 and AS-523, and in contrast to WT cells and IR transformants, there were on average 52±4% molecules of DT that were not synthesized during the light treatment. Indeed, the amount of DT molecules synthesized during the light treatment (1.3–1.4 mol/100 mol Chl *a*, table 3) was close to that synthesized by the LL cells during the same light treatment (1.1 mol/100 mol Chl *a*, table 2) and clearly lower than the amount in HL WT cells (4.2 mol/100 mol Chl *a*) and IR cells (2.3–2.4 mol/100 mol Chl *a*). A difference of 1 mol DT/100 mol Chl *a* can have a significant impact on the extent of NPQ depending on the light treatment (Fig. 5) [2], [9]. Thus in AS transformants there was no direct correlation (see Fig. 6B) between the increase of the DD+DT pool size and the increase of the amount of DT synthesized during the light treatment as observed in WT and IR-5 cells and as usually reported in *P. tricornutum* and other species [25], [44], [49], [51], [52].

Taken together, our results seem to be consistent with the assumption that high irradiance likely enhances the silencing efficiency by inducing the *FcpA* promoter. Surprisingly this effect, although not being strong, was only visible in the AS transformants while the silencing effect appeared to be clearly similar in the IR transformants independent of the irradiance. It could result from a difference between AS and IR silencing mechanisms [35]. Nevertheless, while there is no reason to look out for a differential balance of lipids (namely MGDG and SQDG) in AS an IR HL acclimated cells that would infer on the DD de-epoxidation efficiency [4], a difference of concentrations of the DDE, and/or VDL and/or VRD enzymes as hypothesized above might be possible. For instance, a higher VDL and/or VDR content in the IR transformants might compensate the increased efficiency of the *Vde/Dde* gene silencing under HL and may explain the higher synthesis of DT in IR transformants compared to the AS transformants. Although we are aware that the measurement of the DES and of the amount of DT was an indirect way for assessing the concentration of DDE and thus the silencing efficiency of the *Vde/Dde* gene, our observation might be of importance and would need further thorough investigation.

### Conclusion

The production and the characterization of transformants of *P. tricornutum* by silencing of the gene encoding for the de-epoxidase (VDE/DDE) provides a strong support to the following features of the photoprotective XC and NPQ in diatoms: 1) the *in vivo* demonstration that the VDE uses DD as a substrate which was previously known only from *in vitro* studies [17], it further supports previous works on the special biosynthetic pathway of xanthophylls in diatoms [14], [53]; 2) the *in vivo* demonstration of the tight and specific link between the irradiance, probably the dependent build-up of the transthylakoidal ΔpH, the DD de-epoxidation, the ‘activation’ of DT molecules and the development of NPQ in the mechanistic model proposed for NPQ in diatoms [2], [9] as well as the possible reasons for the discrepancy observed under some conditions of light between these regulatory partners using potentially unspecific inhibitors [18], [19], [24]; 3) a solid basis for hypotheses about the potential functional role of the de-epoxidases related to the VDE/DDE, the VDL and VDR enzymes, their localization within the chloroplast, their role in the DD de-epoxidation and DT synthesis, and their regulation as a function of the transthylakoidal ΔpH.

The results also indicate different silencing efficiencies of antisense (AS) and inverted repeat (IR) DDE transformants at elevated light intensities suggesting the presence of different silencing pathways, e.g. transcript degradation and/or translational inhibition, in diatoms. Investigating the differences between AS and IR silencing mechanisms might help to identify the molecules involved in targeted down-regulation of transcription in algae with secondary plastids. It will provide us with another tool to further investigate the biology and ecology of diatoms on a molecular level.

## Materials and Methods

### Cell Cultivation and Preparation for Physiological Measurements

*P. tricornutum* (University of Texas Culture Collection, strain 646 [64]) wildtype and transformed cells were grown photoautotrophically in sterile 50% artificial seawater f/2 medium [65]. Cultures of 200 mL were incubated at 21°C in airlifts continuously flushed with sterile air. They were illuminated at a light intensity of 45 or 135 µmol photons⋅m^−2^⋅s^−1^ (respectively ‘low light-LL’ and ‘high light-HL’) with white fluorescent tubes (OSRAM, Munich, Germany) in a 16∶8 h light:dark (L:D) cycle. Cells were harvested during the exponential growth phase, 2±0.5 h after the onset of light in the morning. Specific growth rates, μ (d^−1^), were calculated from regression of natural logarithm of culture chlorophyll *a* (Chl *a*) concentration or fluorescence during the exponential growth phase of acclimated cultures.

### PCR and Construction of Plasmids

PCR was performed with a Gradient Master Cycler (Eppendorf, Hamburg, Germany) using recombinant *Pfu* polymerase (Fermentas, St.Leon-Rot, Germany) according to the manufacturer’s instructions. All antisense (AS) fragments were amplified from the N-terminal, less conserved part of the diadinoxanthin de-epoxidase (*Dde*) gene in order to avoid any unspecific hybridization of antisense transcripts with transcripts of the de-epoxidase like or de-epoxidase related genes (see [15]). The fragments have been inserted into the *P. tricornutum* transformation vector pPha-T1 [66], which allows transformation of the diatom selecting for positive transformants using Zeocin™ (Invitrogen, Carlsbad, CA, USA). The 198 bp *Dde* antisense fragment was amplified using the primers DDE-198AS-HindIII-5′ and DDE-198AS-BamHI-3′, the 523 bp fragment was amplified using the primers DDE-523AS-HindIII-5′ and DDE-523AS-BamHI-3′ (see table S1). Inserts were ligated in antisense orientation into the plasmid downstream of the *FcpA* promoter, resulting in pDDE-AS198 and pDDE-AS523, respectively. To construct the pDDE-IR (inverted repeat) plasmid several subsequent cloning steps were performed. First a 293 bp fragment of the *Dde* gene was amplified using the primers DDE-293AS-HindIII-5′ and DDE-523AS-BamHI-3′ and cloned in pPha-T1 in antisense orientation resulting in pPha-T1 AS293. In the following steps a fragment containing the *FcpA* 5′ untranslated region (UTR) downstream of the *FcpA* promoter followed by *eGFP* was amplified from the pPha-T1-GFP vector [67] using the primers SgfI-NcoI-pTV-MCS-5′ and pTV-MCS-BamHI-PmeI-3′. Further a 523 bp long fragment of *Dde* was amplified using the primers SgfI-EcoRI-DDE-523-5′ and DDE-523-NcoI-PmeI-3′. Both fragments were ligated in the pF1-A directional cloning vector (Promega, Madison, WI, USA) according to the manufacturer’s protocol. The resulting vectors were termed pF1-A DDEnc S and pF-1A pTV-MCS. pF1-A DDEnc S was digested with *Ecl136*II and *Fsp*I, pF-1A pTV-MCS was digested with *Pvu*II and *Fsp*I. For both plasmids the half containing the respective insert was separated by horizontal gel-electrophoresis and eluted from the agarose gel. Both parts were ligated, thus giving rise again to the complete pF1-A vector, which contains the 523 bp *Dde* fragment and the 5-UTR-*eGFP* construct, now ligated together. The resulting *Dde* (sense)-5′-UTR-*eGFP* insert was amplified from the plasmid using the primers DDE-523AS-HindIII-5′ and pTV-MCS-BamHI-PmeI-3′. The amplicon was subsequently digested with *BamH*I. After digesting the plasmid pPha-T1 AS293 with *EcoR*V and *BamH*I the digested amplicon was ligated into the plasmid, thus giving rise to the final transformation vector pDDE-IR.

### Biolistic Transformation

Cells were bombarded using the Bio-Rad Biolistic PDS-1000/He Particle Delivery System (Bio-Rad Laboratories, Hercules, Canada) fitted with 1350 psi rupture discs. Tungsten particles (0.7 µm median diameter) were coated with 5 µg of plasmid DNA in the presence of CaCl_2_ and spermidine, as described by the manufacturer. One hour prior to bombardment approximately 10^8^ cells were spread in the center of a plate containing 20 mL of solid culture medium. The plate was positioned at the second level within the biolistic chamber for bombardment. Bombarded cells were allowed to recover for 24 h before being suspended in 1 mL of sterile 50% artificial seawater medium. 250 µL of this suspension were plated onto solid medium containing 50 µg/mL Zeocin™. The plates were incubated at 20°C under constant illumination (40 µmol photons⋅m^−2^⋅s^−1^) for three weeks.

### Isolation of RNA and cDNA Synthesis

Cells were harvested by brief centrifugation (1 min at 2800 g) at room temperature. The pellet was immediately shock-frozen in liquid N_2_. Cell pellets were homogenized by grinding the frozen pellets under liquid N_2_. 1 mL Trizol® (Invitrogen, Carlsbad, CA, USA) was added to the deep frozen grinded powder, which was further homogenized in subsequent steps by vortexing and shaking the solution at room temperature. After adding 200 µL of chloroform, rigorous mixing and centrifugation at 4°C, the aqueous phase was transferred into pre-cooled tubes. After adding 1 volume of ethanol (70%) the solution was transferred into RNeasy® RNA purification columns (Qiagen, Hilden, Germany). Subsequent RNA purification steps were performed according to the manufacturer’s instructions using the provided RW1 and RPE buffers. Although both Trizol® and the RNeasy® purification are designed to remove genomic DNA from the RNA extract, we additionally treated aliquots of the extracted RNA with Turbo™-DNase (Ambion, Woodward, TX, USA). The obtained genomic DNA-free RNA was reverse transcribed using the reverse transcriptase provided by the QuantiTect® reverse transcription Kit (Qiagen, Hilden, Germany). Complete removal of genomic DNA from RNA samples was verified by PCR amplification of intergenic regions after cDNA synthesis. The resulting genomic DNA free cDNA was further used for real-time PCR assays.

### Real-time PCR Assays

Real-time PCR was performed using the Real-Time PCR System 7500 (Applied Biosystems, Lincoln, CA, USA). The following program was utilized for all genes: 10 min of pre-incubation at 95°C followed by 40 cycles for 15 s at 95°C and 1 min at 60°C. Individual real-time PCR reactions were carried out in 20 µL volumes in 96-well plates using Power SYBR® Green PCR Master Mix and optical covers by Applied Biosystems. For amplification of the target (*Dde*) fragment the primers RT-DDE-629-fw and RT-DDE-729-rev (see table S1) were used, the endogenous control fragment (*Gapdh*) was amplified using the primers RT-GapDH-775-fw and RT-GapDH-875-rev. All samples were analyzed in six replicates per experiment and each experiment was repeated independently at least twice. At the end of each reaction, the cycle threshold (Ct) was manually set at the level that reflected the best kinetic PCR parameters, and melting curves were acquired for analysis. The ΔΔCt method [68] was used to analyze the generated data and to calculate relative *Dde* transcript quantities. Wildtype cDNA was used as calibrator and the *Gapdh* gene served as endogenous control. All performed experiments were repeated using the *Actin* gene as endogenous control (primers RT-Actin-24-fw and RT-Actin-124-rev) in order to confirm the obtained results.

### RNaseIII Assays

In order to distinguish between the amounts of single-stranded (ss) and double-stranded (ds) *Dde* transcript quantities determined by real-time PCR, an aliquot of each RNA extract was treated with ShortCut RNase III (NEB, Ipswitch, MA, USA) according to the manufacturer’s instructions. RNase III converts long double-stranded RNA into a heterogeneous mix of short (18–25 bp) RNAs which cannot serve as template for real-time PCR. The digested RNA samples were reverse transcribed and the resulting cDNA analyzed via real-time PCR.

### Pigment Extraction and Analysis

Chl *a* concentrations were determined spectroscopically using the extinction coefficients described by [47]. Cell counts were performed with a Thoma hematocymeter in order to determine the amount of Chl *a* per cell. Pigment analyses were performed by HPLC as described by [69]. After light treatment, 1 mL of cells was sampled and the pigment extracted in a 4°C cold methanol/ammonium acetate (90/10, v/v):ethyl acetate (90∶10, v/v) mix. The xanthophyll de-epoxidation degree (DEP degree in%) was calculated as (DT/DD+DT) × 100 where DD is diadinoxanthin, the epoxidized form and DT is diatoxanthin, the de-epoxidized form. The light treatments were performed as previously described [22] in a 2 mL Clark oxygen electrode vial (model DW1, Hansatech Ltd., England) which was temperature (21°C) controlled. White light of different light intensities was provided by a KL 1500 LCD lamp (Schott, Mainz, Germany) linked to the vial by an optic guide. The cells were continuously stirred during the light exposure to homogenize the suspension. Dithiothreitol (DTT) (Sigma-Aldrich, Munich, Germany) was added from a freshly prepared solution to the cell suspension (20 µM final concentration) and at the beginning of the 15 min dark period preceding the light treatment (according to [18]).

### Chlorophyll Fluorescence Yield

Chl *a* fluorescence yield was monitored with an Imaging-PAM (Walz, Effeltrich, Germany). Fluorescence was excited by a very weak (non-actinic) modulated 470 nm light beam. After 15 min dark-adaptation, continuous actinic light of adjustable intensity was applied in two ways: i) *Light curves*: the irradiance was gradually increased from 0 to 1600 µmol photons⋅m^−2^⋅s^−1^ through 11 steps of 100 s each, ii) *Time kinetics*: the irradiance was fixed at 450 µmol photons⋅m^−2^⋅s^−1^ and the cells illuminated for 5 min. 800 ms pulses of saturating blue light (2800 µmol photons⋅m^−2^⋅s^−1^) were used in order to monitor the evolution of maximal fluorescence during actinic light exposure either at the end of each light step for the light curves or each 30 s for the time kinetics. The average fluorescence measured during the pulses was taken as F_m_ or F_m_′ for dark-adapted and illuminated cells, respectively. For each experiment, the cells were concentrated in the dark as a homogenized cell layer to a final Chl *a* concentration of 20 µg Chl *a*·mL^−1^ (see [70]) on a Millipore A20 paper prefilter. It was controlled that the prefilter was kept wet during the measurement. It was verified that neither the sampling time (2/3 h to 8 h after the onset of light), neither the light period used for growing the cells (16∶8 h L:D or 24∶0 h L:D) nor the length of the dark-adaptation of cells previous to the measurements (from 20 min to several hours) had any influence on the results. Standard fluorescence nomenclature was used. F_0_ and F_m_ are defined as the minimum photosystem (PS) II fluorescence yield of dark-adapted cells and the maximum PSII fluorescence yield reached in such cells during a saturating pulse of white light, respectively. The maximum photosynthetic efficiency of PSII is F_v_/F_m_ where F_v_  =  F_m_−F_0_. The non-photochemical fluorescence quenching is NPQ  =  F_m_/F_m_′−1, where F_m_′ is the maximum PSII fluorescence yield of light-adapted cells. Light curves were fitted with the model of Eilers and Peeters in order to extract rETR_max_ (maximal relative rate of linear electron transport), α (maximum light use efficiency which is the slope of the beginning of the light curve), and E_m_ (light intensity for reaching rETR_max_). rETR_max_  =  ΦPSII × PFD × α × 0.5, where ΦPSII is the effective PSII quantum yield for photochemistry ( =  (F_m_′−F′)/F_m_′) and PFD (Photon Flux Density) is the irradiance in µmol photons⋅m^−2^⋅s^−1^.

## Supporting Information

Figure S1 **Non-photochemical quenching of chlorophyll fluorescence (NPQ) in false color in the WT and two** ***Dde*** **transformants (AS-523 and IR-5) of** ***P. tricornutum*** **cells as measured with an Imaging-PAM (Walz, Germany). Cells were grown as colonies on an agar plate.** Light conditions were: 5 min at 450 µmol photons⋅m^−2^⋅s^−1^. The numbers (from 0 to 1) refer to the NPQ value divided by 4.
(TIF)

Table S1 **Primers used for the construction of transformation vectors and for qPCR measurements.** Small letters in primer sequences indicate the conferred nucleotide substitutions.
(DOC)

Table S2 **Relative quantification of** ***Dde*** **transcripts via real-time PCR.** Transcript levels are relative to the WT and normalized to *Gapdh* expression. Values are average of at least two replicates. total RNA, untreated RNA used for qPCR; ssRNA, single stranded RNA (total RNA treated with RNaseIII); RQ, relative quantity.
(DOC)
