# Supplementary figures and images for: Silencing of the Violaxanthin De-Epoxidase Gene in the Diatom Phaeodactylum tricornutum Reduces Diatoxanthin Synthesis and Non-Photochemical Quenching
Source: PLoS One. 2012 May 18;7(5):e36806. doi: 10.1371/journal.pone.0036806 (PMC3356336; doi:10.1371/journal.pone.0036806)

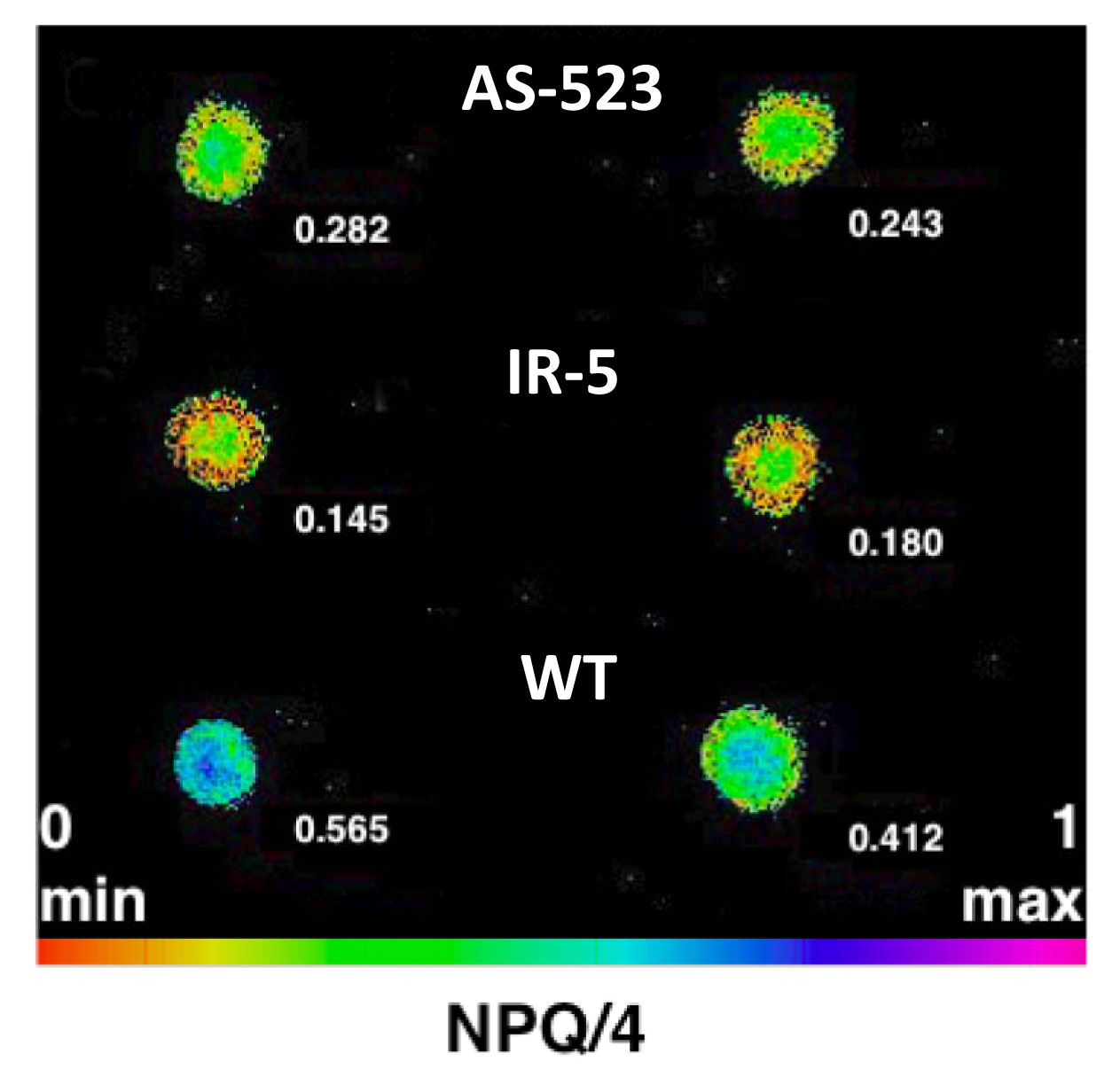

Supplement: Figure S1 — Non-photochemical quenching of chlorophyll fluorescence (NPQ) in false color in the WT and two Dde transformants (AS-523 and IR-5) of P. tricornutum cells as measured with an Imaging-PAM (Walz, Germany). Cells were grown as colonies on an agar plate. Light conditions were: 5 min at 450 µmol photons⋅m−2⋅s−1. The numbers (from 0 to 1) refer to the NPQ value divided by 4. (TIF) [file pone.0036806.s001.tif]
