# Supplementary material for: Silencing of the Violaxanthin De-Epoxidase Gene in the Diatom Phaeodactylum tricornutum Reduces Diatoxanthin Synthesis and Non-Photochemical Quenching
Source: PLoS One. 2012 May 18;7(5):e36806. doi: 10.1371/journal.pone.0036806 (PMC3356336; doi:10.1371/journal.pone.0036806)
Supplement: Table S1 — Primers used for the construction of transformation vectors and for qPCR measurements. Small letters in primer sequences indicate the conferred nucleotide substitutions. (DOC) [file pone.0036806.s002.doc]

**Table S1: Primers used for the construction of transformation vectors and for qPCR measurements. Small letters in primer sequences indicate the conferred nucleotide substitutions.**

| Primer name | Primer sequence (5'-3') |
| --- | --- |
| DDE-198AS-HindIII-5' | GACAAgCttAATCCCTCGTACGGACTTCAGC |
| DDE-198AS-HindIII-3' | aaaaggatcccccccccACGGGAGAGTGACTGGTGTCG |
| DDE-523AS-HindIII-5' | AAAAAGcttCAACCAAAATCCCTCG |
| DDE-523AS-BamHI-3' | TTAATGgAtcCAACGTTGGCGAGGCAC |
| DDE-293AS-HindIII-5' | TCTCGGGCTCCAAgcTTGTCGCTCCGC |
| SgfI-NcoI-pTV-MCS-5' | aaaagcgatcgccaTGgCTGCAAGATCAGCTGGCCTAGC |
| pTV-MCS-BamHI-PmeI-3' | aaaacaaatttggaTCCCTGGTTGAGTTCGATAGCACG |
| SgfI-EcoRI-DDE-523-5' | aaaagcgatcgccgAAttcGACAACCAAAATCCCTCGTACGGAC |
| DDE-523-NcoI-PmeI-3' | aaaacaaatttgccATGgAAACAACGTTGGCGAGGCACTTGGG |
| RT-DDE-629-fw | ACATCTCAGCCGGACAAAACA |
| RT-DDE-729-rev | CCAATTCAGTTTGCCGAAGAAC |
| RT-GapDH-775-fw | ACGGCCGATGTTTCTATGGT |
| RT-GapDH-875-rev | ATCGGTCCTTCTGACGCCTT |
| RT-Actin-24-fw | TGAGACCTTCAATGTCCCGG |
| RT-Actin-124-rev | CATCGCCTGAGTCGAGAACAC |
