# Supplementary material for: Silencing of the Violaxanthin De-Epoxidase Gene in the Diatom Phaeodactylum tricornutum Reduces Diatoxanthin Synthesis and Non-Photochemical Quenching
Source: PLoS One. 2012 May 18;7(5):e36806. doi: 10.1371/journal.pone.0036806 (PMC3356336; doi:10.1371/journal.pone.0036806)
Supplement: Table S2 — Relative quantification of Dde transcripts via real-time PCR. Transcript levels are relative to the WT and normalized to Gapdh expression. Values are average of at least two replicates. total RNA, untreated RNA used for qPCR; ssRNA, single stranded RNA (total RNA treated with RNaseIII); RQ, relative quantity. (DOC) [file pone.0036806.s003.doc]

**Table S2: Relative quantification of *Dde* transcripts via real-time PCR. Transcript levels are relative to the WT and normalized to *Gapdh* expression. Values are average of at least two replicates. total RNA, untreated RNA used for qPCR; ssRNA, single stranded RNA (total RNA treated with RNaseIII); RQ, relative quantity.**

|  | Sample | RQ | RQ_min_ | RQ_max_ |
| --- | --- | --- | --- | --- |
| total RNA |  |  |  |  |
|  | WT | 1.00 | 0.80 | 1.26 |
|  | AS 198-1 | 7.35 | 6.08 | 8.90 |
|  | AS 523-1 | 9.99 | 8.31 | 12.03 |
|  | AS 523-2 | 6.20 | 4.81 | 8.00 |
|  | IR 4 | 1.49 | 1.16 | 1.94 |
|  | IR 5 | 0.76 | 0.55 | 1.07 |
| ss RNA |  |  |  |  |
|  | WT | 1.00 | 0.63 | 1.58 |
|  | AS 198-1 | 1.96 | 1.45 | 2.64 |
|  | AS 523-1 | 0.66 | 0.35 | 1.30 |
|  | AS 523-2 | 0.64 | 0.43 | 0.96 |
|  | IR-4 | 0.81 | 0.58 | 1.15 |
|  | IR-5 | 1.34 | 0.88 | 2.04 |
